# Interaction between Genetic Risks and Socioeconomic Factors on Thyroid Cancer: Evidence from 0.5 Million UK Biobank Participants

**DOI:** 10.3390/cancers15205028

**Published:** 2023-10-18

**Authors:** Yu Li, Yongle Zhan, Wei Mao, Baoxin Wang, Pin Dong, Rong Na

**Affiliations:** 1 Department of Otolaryngology Head and Neck Surgery, Shanghai General Hospital, Shanghai Jiao Tong University School of Medicine, Shanghai 200080, China; 2 Department of Surgery, LKS Faculty of Medicine, The University of Hong Kong, Hong Kong, China

**Keywords:** socioeconomic factors, genetics, interaction, thyroid cancer

## Abstract

**Simple Summary:**

The interaction effect between genetic risk and socioeconomic factors on thyroid cancer remains unclear. In this study, we utilized a large-scale population dataset to comprehensively estimate the independent effects of genetic and socioeconomic factors and their interaction with thyroid cancer (TCa). The results of this study showed that (1) telomerase reverse transcriptase (*TERT*) variants significantly related to TCa risk were commonly situated in the intron 2 region; and (2) low-to-medium genetic risk combined with low household income was associated with a high TCa risk, whereas medium-to-high genetic risk combined with a higher education level and frequent social connection was associated with an increased TCa risk. These findings furnish insights into risk stratification and are informative for implementing the precise screening of thyroid cancer in the general population.

**Abstract:**

Background: There is a research gap between genetic predisposition, socioeconomic factors, and their interactions on thyroid tumorigenesis. Methods: Individual and genetic data were obtained from UK Biobank. Logistic regression models were used to evaluate the association between genetic risk, socioeconomic factors, and thyroid cancer (TCa). A stratified analysis was conducted to estimate their joint effects. A two-sample Mendelian randomization (MR) analysis was further used to examine the potential causality. Results: A total of 502,394 participants were included in this study. Three index loci (rs4449583, rs7726159, and rs7725218) of telomerase reverse transcriptase (*TERT*) were found to be significantly related to incident TCa. Association analyses showed that high genetic risk, low household income, and high education level were independent risk factors, while unemployment and frequent social connection were suggestive risk factors for TCa. Interaction analyses showed that in participants with low genetic risk, low household income was significantly associated with TCa (odds ratio [OR] = 1.56, 95% confidence interval [CI]: 1.00–2.46). In participants with high genetic risk, those with a high education level (OR = 1.32, 95%CI: 1.06–1.65) and frequent social connection (OR = 1.36, 95%CI: 1.02–1.81) had a significantly increased risk of TCa. However, no causal relationship was observed in the MR analysis. Conclusion: Interactions exist between genetic risk, household income, education level, and social connection and thyroid cancer.

## 1. Introduction

Thyroid cancer (TCa) is one of the most frequent endocrine tumors, with an estimated 586,000 new cases and 44,000 cancer deaths worldwide in 2020, according to Global Cancer Statistics [1]. A wide range of risk factors for TCa have been identified to date [2], such as female sex, radiation exposure, excessive iodine intake, comorbid autoimmune disease, socioeconomic factors, and genetic susceptibility.

Genetic predisposition is the most critical risk factor for thyroid tumorigenesis. People with a family history of TCa were reported to have a three- to five-fold lifetime increment of TCa risk [3]. A prior study using UK Biobank (UKB) data of 264,956 participants suggested that compared to people with low genetic risk, based on single nucleotide polymorphisms (SNPs), those with intermediate and high risks were associated with 71% and 125% increased risks of incident TCa [4]. Mutations in telomerase-related gene regions in relation to tumorigenesis have long intrigued researchers. An example is telomerase reverse transcriptase (*TERT*), a key determinant of the enzymatic activity of telomerase, whose mutation was found to be related to numerous cancers, such as breast, bladder, prostate, and thyroid cancers [5,6].

In addition to genetic risk, socioeconomic factors have been reportedly associated with TCa development. A population-based study including one million participants showed that TCa incidence was 1.5 times elevated in high human development index (HDI) regions, compared with low HDI regions [7]. However, other observational studies from Europe and East Asia held the opposite view that higher income level was related to lower TCa risk [4,8]. In our prior research using the Surveillance, Epidemiology, and End Results Database (SEER) [9], we found that patients insured by Medicaid had a 2.15-fold poorer cancer-specific survival (CSS) and a 2.42-fold poorer overall survival (OS) than those insured by commercial insurance or Medicare. Additionally, divorced or widowed status, rural living location, and low Yost index were significantly associated with poor CSS and OS of thyroid adenomas/adenocarcinomas.

Although genetic and socioeconomic factors have been proven to influence TCa independently, the interaction effect between them remains unclear. Only one study to date has reported an interplay between genetic susceptibility and social behaviors on thyroid cancer [10]. Socioeconomic factors are suggested to play a crucial role in modifying the tumorous biological determinants activated by genetic mutations [11]. For instance, Goel et al. observed a significant synergistic effect between genetic variation and low neighborhood socioeconomic status for breast carcinogenesis [12]. Given a literature gap and equivocal understanding of the gene–environmental interaction on TCa etiology, it is essential to investigate how genetics and socioeconomics interplay in thyroid tumorigenesis.

Therefore, in this study, we utilize the latest UKB data to (1) comprehensively explore the genetic predisposition of TCa, including aggregate genetic risks and *TERT* loci polymorphisms; (2) re-confirm the risk effect of common socioeconomic factors (income, education, employment, and social connection) on TCa development; and (3) explore the interaction between the aforementioned genetic and socioeconomic factors and thyroid tumorigenesis.

## 2. Materials and Methods

### 2.1. Study Population

The study was performed using the UK Biobank [13], a large-scale biomedical database containing up to 0.5 million individuals between 40 and 69 years old recruited from 2006 to 2010. Participants were followed up for thyroid cancer (C73) using records linkage with the regional system of disease surveillance, chronic disease management, and electronic health records (EHRs) based on diagnostic codes from the International Classification of Diseases, 10th revision (ICD-10).

### 2.2. Measurement of Socioeconomic Factors

Demographic information including age and sex was collected by questionnaire. All societal exposures were derived from the baseline assessment center data collection, including annual household income, age finishing full-time education, highest education level, employment, household size, frequency of friend/family visits, and frequency of confiding in others. Missingness of variables of interest are shown in Table S1.

### 2.3. Ascertainment of Genetic Risk

Blood samples of each participant were collected, and germline DNA samples were extracted via a whole blood genomic DNA extraction kit and then further genotyped using the UK Biobank Axiom array [14]. Imputation for UKB genotyping data was performed using the IMPUTE4 program [14]. Genetic loci (SNPs) in relation to TCa were collected from the latest genome-wide association study (GWAS) [15] (Table S2), and these SNPs were used for calculating the polygenic risk score (PRS) in the UKB dataset, which is the proxy of TCa heritability.

### 2.4. Two-Sample Mendelian Randomization Study

A two-sample Mendelian randomization (MR) study was conducted to examine the causal effects of socioeconomic factors on thyroid cancer. This methodology, based on instrumental variable (IV) principles, allowed us to explore the causality between exposures and outcomes using multiple genetic variants from summary-level data [16]. This approach has been widely used to explore the risk factors for various tumors [17,18]. The framework of this study was in line with the three MR assumptions: (i) instrumental variables (SNP) were truly associated with the socioeconomic factors, (ii) SNPs were unrelated to the confounders on the exposure–TCa nexus, and (iii) SNPs affected TCa only through socioeconomic factors. The instrumental variables (SNPs) of the socioeconomic factors were obtained from publicly downloadable sources, including the GWAS catalog (www.ebi.ac.uk/gwas (accessed on 26 July 2023)) and the MR-Base repository of full GWAS association statistics (www.mrbase.org (accessed on 26 July 2023)). Most of the summary GWAS data on the exposure traits were from the UKB database. To reduce bias caused by sample overlap between the exposure and the outcome datasets, we used the FinnGen r9 database to collect the summary GWAS data on TCa traits [19] (Table S3).

### 2.5. Statistical Analysis

The association between each SNP and TCa risk was estimated by the odds ratio (OR), 95% confidence interval (CI), and corresponding *p*-value using logistic regression analysis with adjustment for age and sex based on an additive model. We further used the MAGMA v1.10 software to implement the gene-based analysis according to the remission and percentage improvement in GWAS *p*-values [20]. The gene-based analysis was performed based on genetic variants and linkage disequilibrium (LD) in the 1000 Genomes European panel reference datasets, and then SNPs were assigned to genes using the MAGMA NCBI37.3.gene.loc file with a 10 kb window. Associations were estimated using Z statistics and corresponding *p*-values. The PRS was calculated by aggregating the number of risk alleles carried in each individual, with effect size weighted for each variance. Regional plots and an LD heatmap were created by using LocusZoom (http://csg.sph.umich.edu/locuszoom/ (accessed on 26 June 2023)) and LDBlockShow v1.39 software [21]. Since the percentages of missing values were less than 2% for all variables of interest, all association and stratified analyses below were complete case analyses. Risk stratification of thyroid cancer was conducted using a tertile method, and participants were divided into low, medium, and high genetic risks correspondingly. Joint analyses of genetic and socioeconomic factors were performed by creating categorical interaction terms, which were further examined by Wald tests. The random-effects inverse-variance weighted method was used to pool the effects of proxy SNPs on TCa in the MR main analysis [16]. A two-tailed Bonferroni correction method for *p*-values was adopted in baseline comparison and SNP–TCa association analyses. Other statistical tests were two-tailed, and *p*-values were deemed statistically significant at the <0.05 level. All statistical analyses were performed under PLINK v1.90 and R4.1.2.

## 3. Results

### 3.1. Participant Characteristics

A total of 502,394 participants (1026 TCa cases/501,368 controls) were included in this study (Table 1). Briefly, female sex (*p* < 0.001), low household income (*p* = 0.001), and unemployment status (*p* = 0.004) were significantly observed in TCa patients. These patients were unlikely to be involved in heavy work in their jobs (*p* = 0.026).

### 3.2. Association between TERT SNPs and TCa Risk

**Figure 1:**
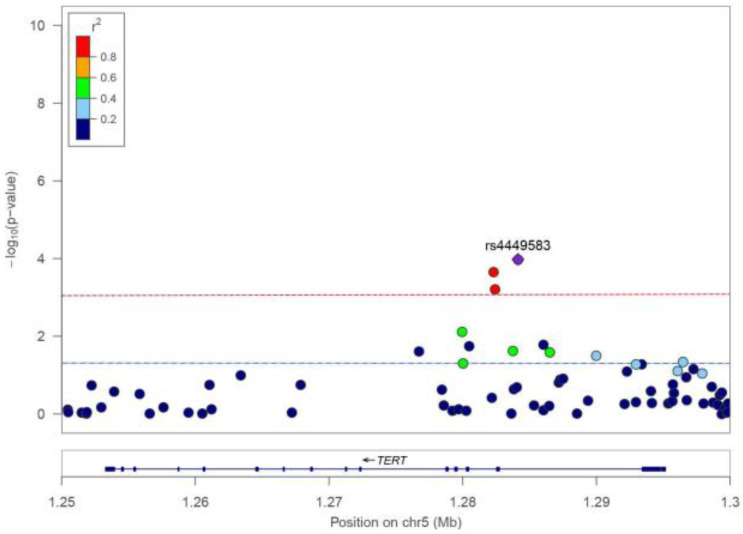
Regional plot of thyroid cancer-related SNPs in the *TERT* locus. (Statistical significance: *p* < 6.41 × 10^−4^ in the red dotted line; suggestive significance: *p* < 0.05 in the blue dotted line; r^2^ refers to the correlation coefficient of the linkage disequilibrium between loci.)

**Figure 2:**
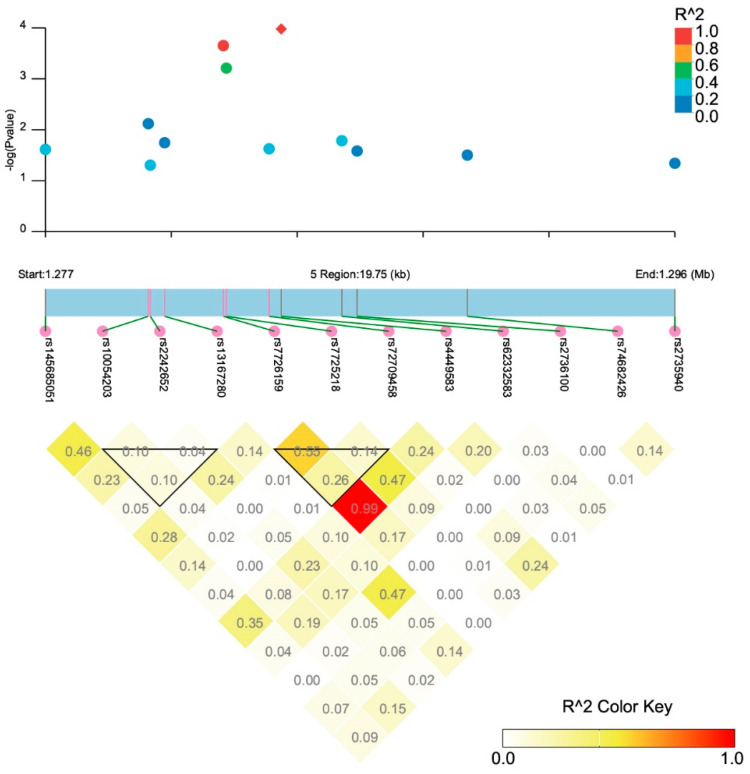
Significant thyroid cancer-related SNPs and their linkage disequilibrium status. (R^2^ refers to the correlation coefficient of the linkage disequilibrium between loci.)

After quality control, 473,367 participants with germline genetic variation data were included in the association analysis on *TERT* SNPs and TCa risk. A total of 12 tagging SNPs were identified (Figure 1), among which rs4449583 (OR = 1.20, 95%CI: 1.10–1.32, *p* = 1.05 × 10^−4^), rs7726159 (OR = 1.19, 95%CI: 1.09–1.31, *p* = 2.23 × 10^−4^), and rs7725218 (OR = 1.18, 95%CI: 1.07–1.29, *p* = 6.17 × 10^−4^) were significantly associated with TCa after Bonferroni correction for multiplicity (*p* < 0.0042) (Table 2). Figure 2 shows that no linkage disequilibrium was observed between the index SNP rs4449583 (located at intron 2 of *TERT*) and other surrounding loci, which indicated this locus to be an independent risk locus for thyroid cancer. We further performed a gene-based analysis using 49 *TERT* SNPs and found that *TERT* was significantly related to thyroid cancer (Z = 2.76, *p* = 0.003).

### 3.3. Genetic Susceptibility, Socioeconomic Factors, and TCa Risk

In terms of genetic susceptibility, compared to participants with low PRS, those with high and medium PRSs had 2.49- (95%CI: 2.10–2.94) and 1.63-fold (95%CI: 1.36–1.95) increased risks of TCa. As for socioeconomic factors, the crude model showed that low annual household income (<£52,000), high education level (college/university or above), unemployed status, and frequent social connection (≥1 time/week) were potential risk factors. After adjustment with age and sex, we found that low household income and high education level were associated with 23% (OR = 1.23, 95%CI: 1.02–1.47) and 19% (OR = 1.19, 95%CI: 1.02–1.39) increased risks of TCa (Table 3).

### 3.4. Interaction between Genetic and Socioeconomic Factors on TCa Risk

Joint analyses showed a potential interplay between genetic risk, household income, and frequent social connection (*p-*_interaction_ < 0.05). After stratification, we observed that in participants with low PRS, low household income was significantly associated with TCa (OR = 1.56, 95%CI: 1.00–2.46). In participants with medium PRS, high TCa risk was also observed in those with low household income (OR = 1.46, 95%CI: 1.03–2.08). In participants with high PRS, those with a high education level (OR = 1.32, 95%CI: 1.06–1.65) and frequent social connections (OR = 1.36, 95%CI: 1.02–1.81) had a significantly increased risk of TCa (Table 4).

To estimate the interaction between *TERT* polymorphism, socioeconomic factors, and TCa risk, we focused on the variant of the index SNP rs4449583. We found that in participants carrying a genotype of CC, those with low household income had a 45% (OR = 1.45, 95%CI: 1.06–1.98) significantly increased risk of TCa, while those with a high education level and unemployment had 25% and 31% increased risks of TCa (Table 5).

### 3.5. Examination of Causal Effect by MR Analysis

An MR analysis was further performed to examine the causal effect between the aforementioned socioeconomic factors and thyroid cancer risk. But we did not observe any significant causality of low family income, high education level, unemployed status, and frequent social connection on incident TCa (*p* > 0.05) (Table 6 and Figures S1 and S2).

## 4. Discussion

This large-scale population-based study comprehensively evaluated the association between genetic susceptibility, socioeconomic factors, and their interactions with thyroid cancer. Specifically, we found that (1) half of the variants significantly related to TCa risk were situated in the intron 2 region of *TERT*; and (2) low-to-medium genetic risk combined with low household income was associated with a high TCa risk, whereas medium-to-high genetic risk combined with high education level or frequent social connection was associated with an increased TCa risk. Results of the present study are informative for risk stratification and the precise screening of thyroid cancer in the general population.

This study suggested that risk loci in the intron 2 region were significantly associated with TCa rather than the promoter region of *TERT*. As reported by previous research, there may be a putative regulatory region located in intron 2 of *TERT* that can elicit biological functions by genome organization, transcription regulation, and alternative splicing, etc. [22]. For instance, a prior functional experiment found that SNP rs2736100 was located in an intronic enhancer and could pose a genotype-specific impact on *TERT* expression by gene regulation [23]. In addition, DNA methylation, often occurring in intron regions [24], plays an important role in tissue-specific transcriptional regulation and is considered a biomarker for multiple cancers [25].

Another important finding of this study is the joint effect between genetic susceptibility and socioeconomic factors. Our study revealed that people with lower family income had a higher risk of TCa, even under low-to-medium genetic risk, whilst people with better social connection combined with high genetic risk should receive regular screening, since they had a higher TCa risk. Household income, regarded as a surrogate marker for health disparity, can influence people when it comes to accessing primary care and health examinations [26]. Other studies showed that a bottom income level was significantly associated with advanced-stage TCa and poor cancer-specific survival [26,27]. The positive correlation between high education level and incident TCa may be due to an early diagnosis [28]. People with a higher level of education have generally increased health awareness, and they tend to seek early medical attention, which may allow them to detect an indolent cancer. Our study is the first to investigate a positive relationship between frequent family/friend visits and the risk of thyroid cancer. A plausible explanation is the risky lifestyle (e.g., excessive iodine intake) aggregation and spread among family members/friends through social network and communication. Another explanation is that these people become more informed about their cancer risk and have increased awareness to receive examinations, resulting in early diagnosis. The findings of the present study provide insights into the risk stratification and precise screening of thyroid cancer in the general population.

There are several limitations in this study. First, limited thyroid cancer cases have been collected in the UK Biobank dataset. However, the prospective cohort design based on a large-scale general population with sufficient genetic data provided strong evidence for assessing the effects of genetic and socioeconomic factors on TCa. Second, given the scant genome-wide association analysis on TCa subtypes, we could not investigate the association between genetic and socioeconomic factors and TCa from a subtype-specific perspective. This merits further exploration in future research. Third, the limited number of thyroid cancer deaths in the current dataset preclude us from exploring the interplay effect on cancer-specific death. Fourth, the test in this study that was based on statistical significance may lead to unimportant differences, simply due to large sample sizes. More attention should be paid to the sizes of point estimates, as well as the deviations between interval estimates and the crossing point. Finally, the residual confounding might bias the effect estimate in the cohort study because we did not observe a causal relationship between socioeconomic factors and TCa in the MR analysis.

## 5. Conclusions

To sum up, this study utilizes large-scale population data from the UK Biobank to comprehensively evaluate the relationship and interaction of genetic susceptibility (including cumulative germline genetic risk and *TERT* variation) and socioeconomic factors with the risk of thyroid cancer. We find that low-to-medium genetic risk combined with low household income is associated with a high TCa risk, whereas a medium-to-high genetic risk combined with high education level or frequent social connection is associated with an increased TCa risk. The findings of this study furnish insights into risk stratification and is imperative for implementing precise screening of thyroid cancer in the general population.

## Figures and Tables

**Table 1:** Characteristics of thyroid cancer cases and controls in the UKB population (n, %).

	Cases	Controls	*p*-Value
**N**	1026	501,368	
**Age**, years (mean ± SD)	56.9 ± 7.7	56.5 ± 8.1	0.170
**Sex**			**<0.001**
Female	781 (76.1)	272,534 (54.4)	
Male	245 (23.9)	228,834 (45.6)	
**Annual household income**			**0.001**
≤£30,999	419 (41.2)	204,912 (41.4)	
£31,000–£51,999	242 (23.8)	110,508 (22.3)	
≥£52,000	180 (17.7)	108,991 (22.0)	
Not known/Refuse to answer	175 (17.3)	70,953 (14.3)	
Missing	10	6004	
**Age finishing full-time education**			0.740
≤15 years	206 (30.7)	103,019 (30.6)	
16–20 years	392 (58.4)	200,877 (59.7)	
≥21 years	60 (8.9)	26,320 (7.8)	
Not known/Refuse to answer	13 (1.9)	6451 (1.9)	
Missing	355	164,701	
**Education level**			0.260
College/university or above	352 (34.6)	160,765 (32.4)	
High school	121 (11.9)	55,186 (11.1)	
Middle school or below	363 (35.7)	190,216 (38.3)	
Not known/Refuse to answer	181 (17.8)	90,568 (18.2)	
Missing	9	4633	
**Employment status**			**0.004**
Employed	537 (52.4)	286,529 (57.2)	
Unemployed	472 (46.1)	209,105 (41.8)	
Not known/Refuse to answer	15 (1.5)	4864 (1.0)	
Missing	2	870	
**Job involves heavy work**			**0.026**
Never/rarely	384 (71.0)	186,824 (64.9)	
Sometimes	97 (17.9)	61,951 (21.5)	
Usually/always	60 (11.1)	38,808 (13.5)	
Not known/Refuse to answer	0 (0.0)	335 (0.1)	
Missing	485	213,450	
**Job involves walking or standing**			0.670
Never/rarely	198 (36.6)	101,236 (35.2)	
Sometimes	168 (31.1)	88,036 (30.6)	
Usually/always	175 (32.3)	98,265 (34.1)	
Not known/Refuse to answer	0 (0.0)	379 (0.1)	
Missing	485	213,452	
**Job involves night shift work**			0.280
Never/rarely	58 (53.2)	25,583 (49.5)	
Sometimes	23 (21.1)	14,583 (28.2)	
Usually/always	28 (25.7)	11,191 (21.6)	
Not known/Refuse to answer	0 (0.0)	343 (0.7)	
Missing	917	449,668	
**Household size**			0.260
One	186 (18.2)	92,701 (18.6)	
Two	506 (49.5)	232,201 (46.5)	
Three or more	326 (31.9)	171,925 (34.4)	
Not known/Refuse to answer	5 (0.5)	2283 (0.5)	
Missing	3	2258	
**Frequency of friend/family visits**			0.057
<1 time/week	194 (19.1)	108,137 (21.8)	
1 time/week	348 (34.2)	176,024 (35.4)	
≥2 times/week	467 (45.9)	209,292 (42.1)	
Not known/Refuse to answer	8 (0.8)	3270 (0.7)	
Missing	9	4645	
**Frequency of confiding in others**			0.082
<1 time/month	182 (17.8)	98,879 (19.8)	
1 time/month to 4 times/week	283 (27.6)	125,673 (25.1)	
≥5 times/week	531 (51.9)	257,973 (51.5)	
Not known/Refuse to answer	28 (2.7)	17,931 (3.6)	
Missing	2	912	

SD, standard deviation. The distributions of baseline characteristics were compared between two groups using a *t*-test for continuous variables, while a chi-square test was used for categorical variables. Significant *p*-value (*p* < 0.05) are shown in bold.

**Table 2:** Significant associations between *TERT* SNPs and thyroid cancer in the UKB.

SNP ID	Position *	Location	Alleles ^#^	RAF	OR (95% CI)	*p*-Value
rs145685051	1276736	Intron 6	G/A	0.017	1.41 (1.05–1.90)	0.024
rs10054203	1279964	Intron 4	C/G	0.399	1.14 (1.03–1.25)	0.008
rs2242652	1280028	Intron 4	A/G	0.189	1.12 (1.00–1.25)	0.049
rs13167280	1280477	Intron 3	A/G	0.119	1.18 (1.03–1.34)	0.018
rs7726159	1282319	Intron 3	A/C	0.327	1.19 (1.09–1.31)	2.23 × 10^−4^
rs7725218	1282414	Intron 3	A/G	0.341	1.18 (1.07–1.29)	6.17 × 10^−4^
rs72709458	1283755	Intron 2	T/C	0.201	1.14 (1.02–1.27)	0.024
rs4449583	1284135	Intron 2	T/C	0.325	1.20 (1.10–1.32)	1.05 × 10^−4^
rs62332583	1286037	Intron 2	T/C	0.014	1.48 (1.07–2.03)	0.016
rs2736100	1286516	Intron 2	C/A	0.503	1.11 (1.01–1.21)	0.026
rs74682426	1289975	Intron 2	A/C	0.133	1.15 (1.01–1.31)	0.031
rs2735940	1296486	Promoter	A/G	0.514	1.10 (1.00–1.20)	0.045

* Located in chromosome 5; ^#^ risk allele/reference allele. SNP, single nucleotide polymorphism; RAF, risk allele frequency; OR, odds ratio; 95% CI, 95% confidence interval.

**Table 3:** Thyroid cancer risks by polygenic risk levels and socio-economic factors.

	Crude Model		Adjusted Model	
	OR (95%CI)	*p*-Value	OR (95%CI)	*p*-Value
**PRS levels**				
Low	1.00		1.00	
Medium	**1.63 (1.36–1.94)**	**<0.001**	**1.63 (1.36–1.95)**	**<0.001**
High	**2.47 (2.09–2.91)**	**<0.001**	**2.49 (2.10–2.94)**	**<0.001**
**Annual household income**				
≥£52,000	1.00		1.00	
<£52,000	**1.27 (1.08–1.50)**	**0.005**	**1.23 (1.02–1.47)**	**0.029**
**Age finishing full-time education**				
20 years or less	1.00		1.00	
21 years or more	1.01 (0.85–1.19)	0.952	1.02 (0.85–1.21)	0.852
**Education level**				
High school or below	1.00		1.00	
College/university or above	**1.15 (1.00–1.32)**	**0.049**	**1.19 (1.02–1.39)**	**0.023**
**Employment status**				
Employed	1.00		1.00	
Unemployed	**1.20 (1.06–1.36)**	**0.003**	1.10 (0.92–1.32)	0.315
**Household size**				
Three or more	1.00		1.00	
Two or less	1.12 (0.98–1.28)	0.084	1.04 (0.90–1.21)	0.586
**Frequency of friend/family visits**				
<1 time/week	1.00		1.00	
≥1 time/week	**1.18 (1.01–1.37)**	**0.039**	1.04 (0.899–1.22)	0.608
**Frequency of confiding in others**				
<1 time/week	1.00		1.00	
≥1 time/week	1.15 (0.98–1.35)	0.083	1.04 (0.88–1.22)	0.650

PRS, polygenic risk score; OR, odds ratio; CI, confidence interval. The adjusted model for PRS–cancer association includes age and sex. The adjusted model for socioeconomics–cancer association includes age, sex, ethnicity, income, education level, employment status, and frequency of friend/family visits. Significant *p*-value (*p* < 0.05) are shown in bold.

**Table 4:** Specific socioeconomic factors in relation to thyroid cancer stratified by levels of polygenic risk score.

PRS Levels	Sample Size	Socioeconomic Factors	OR (95%CI)	*p-*_interaction_
**Annual household income**				
Low	35,664	≥£52,000	1.00	**0.049**
	102,771	<£52,000	**1.56 (1.00–2.46)**	
Medium	35,487	≥£52,000	1.00	
	102,900	<£52,000	**1.46 (1.03–2.08)**	
High	35,820	≥£52,000	1.00	
	103,109	<£52,000	1.03 (0.80–1.32)	
**Education level**				
Low	62,487	High school or below	1.00	0.179
	70,275	College/university or above	1.16 (0.81–1.65)	
Medium	61,932	High school or below	1.00	
	70,740	College/university or above	1.02 (0.77–1.34)	
High	61,810	High school or below	1.00	
	70,510	College/university or above	**1.32 (1.06–1.65)**	
**Employment status**				
Low	93,148	Employed	1.00	0.137
	67,481	Unemployed	1.11 (0.73–1.69)	
Medium	92,695	Employed	1.00	
	67,966	Unemployed	1.15 (0.83–1.59)	
High	92,849	Employed	1.00	
	67,855	Unemployed	1.05 (0.80–1.36)	
**Frequency of friend/family visits**				
Low	35,528	<1 time/week	1.00	**0.001**
	125,397	≥1 time/week	0.70 (0.47–1.04)	
Medium	35,261	<1 time/week	1.00	
	125,749	≥1 time/week	0.94 (0.68–1.31)	
High	34,894	<1 time/week	1.00	
	126,256	≥1 time/week	**1.36 (1.02–1.81)**	

PRS, polygenic risk score; OR, odds ratio; CI, confidence interval. Adjusted model includes age, sex, ethnicity, income, education level, employment status, and frequency of friend/family visits. Significant *p*-value (*p* < 0.05) are shown in bold.

**Table 5:** Interaction between *TERT* polymorphisms of rs4449583 and socioeconomic factors on thyroid cancer.

Genotyping of rs4449583	Sample Size	Socioeconomic Factors	OR (95%CI)	*p-*_interaction_
** Annual household income **				
CC	46,161	≥£52,000	1.00	** 0.006 **
	134,601	<£52,000	** 1.45 (1.06–1.99) **	
CT	45,346	≥£52,000	1.00	
	129,076	<£52,000	1.12 (0.85–1.47)	
TT	11,000	≥£52,000	1.00	
	30,979	<£52,000	1.03 (0.63–1.68)	
** Education level **				
CC	81,281	High school or below	1.00	0.670
	91,088	College/university or above	1.24 (0.97–1.59)	
CT	77,849	High school or below	1.00	
	88,780	College/university or above	1.08 (0.86–1.37)	
TT	18,833	High school or below	1.00	
	21,482	College/university or above	1.30 (0.84–2.00)	
** Employment status **				
CC	120,841	Employed	1.00	0.179
	88,336	Unemployed	1.31 (0.97–1.75)	
CT	116,324	Employed	1.00	
	85,626	Unemployed	1.02 (0.77–1.36)	
TT	28,228	Employed	1.00	
	20,447	Unemployed	0.94 (0.57–1.57)	
** Frequency of friend/family visits **				
CC	45,322	<1 time/week	1.00	0.720
	164,281	≥1 time/week	1.11 (0.81–1.51)	
CT	43,871	<1 time/week	1.00	
	158,583	≥1 time/week	1.09 (0.82–1.46)	
TT	10,565	<1 time/week	1.00	
	38,210	≥1 time/week	1.02 (0.61–1.71)	

*TERT*, telomerase reverse transcriptase; OR, odds ratio; CI, confidence interval. Adjusted model includes age, sex, ethnicity, income, education level, employment status, and frequency of friend/family visits. Significant *p*-value (*p* < 0.05) are shown in bold.

**Table 6:** Genetic causal effects of socioeconomic factors on thyroid cancer risk based on the IVW model.

Phenotype	Instrument			Effect Size		Heterogeneity			Pleiotropy	
	SNPs (n)	F-Stat	R^2^ (%)	OR (95%CI)	*p*	Q-Stat	*p*	I^2^ (%)	MR-Egger Intercept	*p*
Lower income	47	57.12	0.70	1.20 (0.61–2.36)	0.589	59.42	0.089	22.58	0.00	0.945
College/university degree	241	9.05	0.68	0.93 (0.50–1.71)	0.811	262.77	0.150	8.66	0.02	0.051
Unemployed	9	10.48	0.02	235.78 (0.29–19,204)	0.110	7.81	0.452	0.00	−0.03	0.327
Frequent friend/family visits	21	47.70	0.25	1.10 (0.32–3.72)	0.881	28.72	0.093	30.37	0.05	0.413

OR, odds ratio; CI, confidence interval; IVW, inverse-variance weighted; SNP, single nucleotide polymorphism; MR, Mendelian randomization.

## Data Availability

UKB data used in this research are publicly available to qualified researchers upon application to the UK Biobank (www.ukbiobank.ac.uk (accessed on 12 May 2023)). The study protocol, statistical analysis plan, and analytical code of this study will be available from the time of publication in response to any reasonable request to the corresponding author.

## Supplementary Materials

The following supporting information can be downloaded at: https://www.mdpi.com/article/10.3390/cancers15205028/s1. Figure S1. Scatter plots of the four socioeconomic traits in relation to thyroid cancer. Figure S2. Funnel plots of each single nucleotide polymorphism of the four socioeconomic traits in relation to thyroid cancer. Table S1. Missingness of variables of interest in this study (n, %). Table S2. Association results from latest genome-wide association studies for previously reported thyroid cancer risk loci in European ancestry. Table S3. Characteristics of GWAS summary data for socioeconomic factors and thyroid cancer in European ancestry.
