# Supplementary material for: Interaction between Genetic Risks and Socioeconomic Factors on Thyroid Cancer: Evidence from 0.5 Million UK Biobank Participants
Source: Cancers (Basel). 2023 Oct 18;15(20):5028. doi: 10.3390/cancers15205028 (PMC10605197; doi:10.3390/cancers15205028)
Supplement: Supplementary file 1 [file cancers-15-05028-s001.zip › Supplementary Figures.pdf]

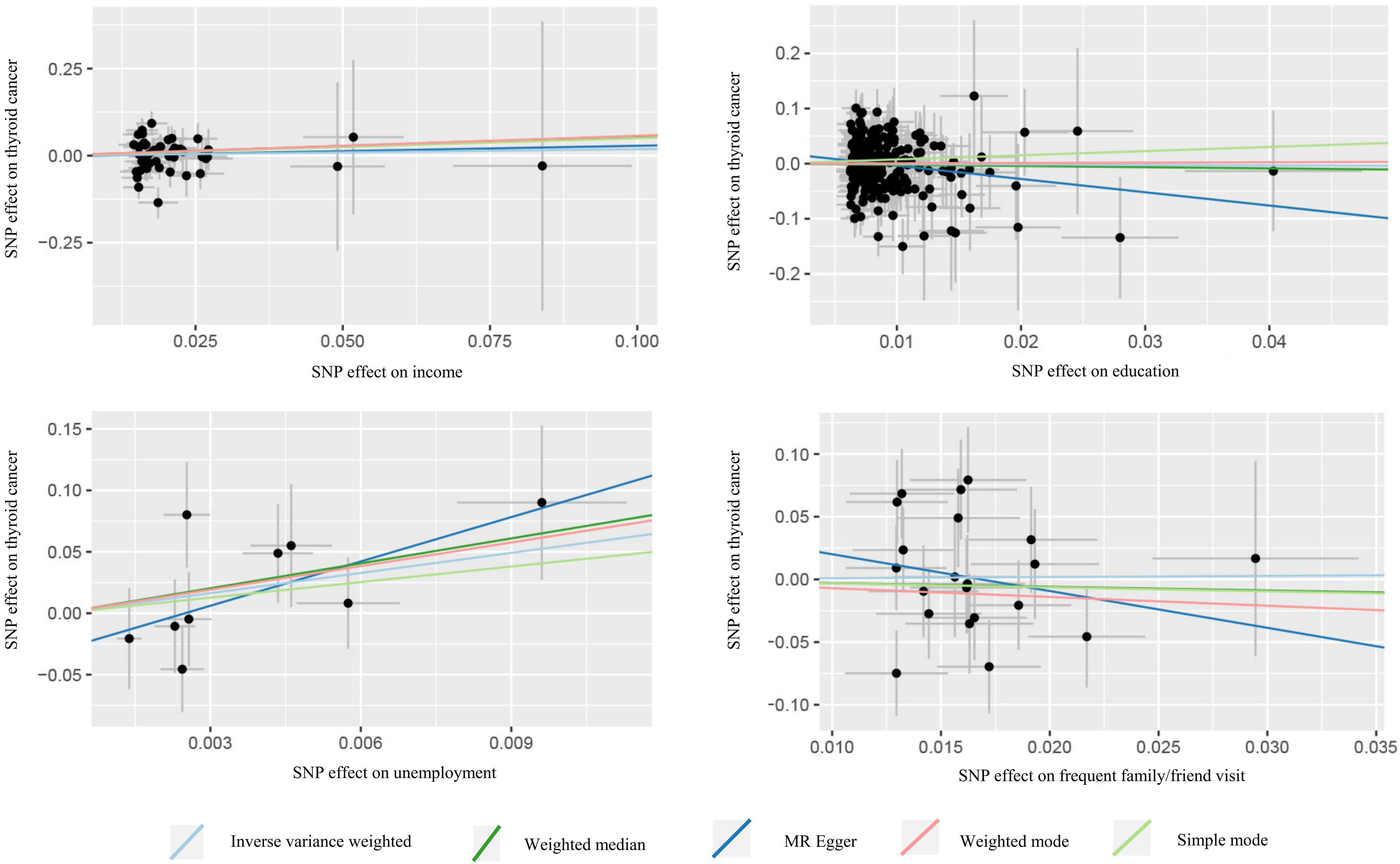

Figure S1. Scatter plots of the four socioeconomic traits in relation to thyroid cancer

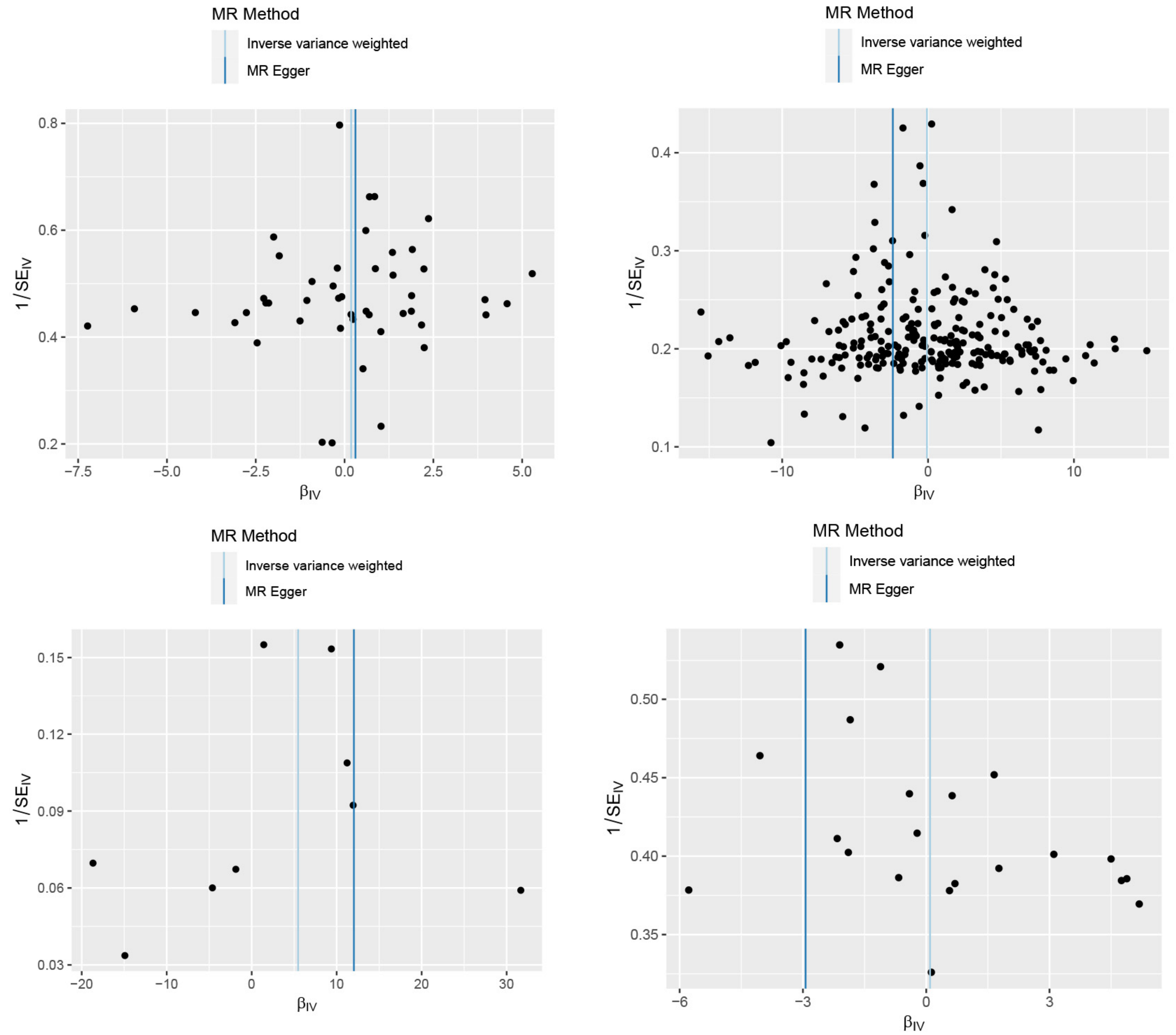

Figure S2. Funnel plots of each single nucleotide polymorphism of the four socioeconomic traits in relation to thyroid cancer
