# Supplementary material for: Interaction between Genetic Risks and Socioeconomic Factors on Thyroid Cancer: Evidence from 0.5 Million UK Biobank Participants
Source: Cancers (Basel). 2023 Oct 18;15(20):5028. doi: 10.3390/cancers15205028 (PMC10605197; doi:10.3390/cancers15205028)
Supplement: Supplementary file 1 [file cancers-15-05028-s001.zip › Supplementary Tables.pdf]

Table S1. Missingness of variables of interest in this study (n, %)

	**Cases (n=1026)**	**Controls (n=501,368)**
Age	0 (0.0)	0 (0.0)
Sex	0 (0.0)	0 (0.0)
Ethnicity	2 (0.2)	892 (0.2)
Annual household income	10 (1.0)	6,004 (1.2)
Education level	9 (0.9)	4,633 (0.9)
Employment status	2 (0.2)	870 (0.2)
Household size	3 (0.3)	2,258 (0.5)
Frequency of friend/family visits	9 (0.9)	4,645 (0.9)
Frequency of confiding in others	9 (0.9)	912 (0.2)

Table S2. Association results from latest genome-wide association studies for previously reported thyroid cancer risk loci in European ancestry

SNP	Locus	Effect allele	OR	LCI	UCI	*P* value
rs12129938	1q42.2	A	1.32	1.20	1.43	4.00×10^{-11}
rs11693806	2q35	C	1.43	1.33	1.54	1.50×10^{-24}
rs966423	2q35	C	1.28	1.20	1.37	8.30×10^{-15}
rs6759952	2q35	T	1.25	1.16	1.34	6.40×10^{-10}
rs6793295	3q26.2	T	1.23	1.15	1.33	2.70×10^{-08}
rs10069690	5p15.33	T	1.20	1.12	1.29	3.20×10^{-07}
rs73227498	5q22.1	A	1.37	1.23	1.49	3.00×10^{-10}
rs2466076	8p12	G	1.32	1.23	1.41	1.50×10^{-17}
rs2439302	8p12	G	1.32	1.23	1.41	5.70×10^{-17}
rs1588635	9q22.33	A	1.70	1.59	1.82	2.00×10^{-58}
rs965513	9q22.33	A	1.70	1.59	1.79	2.20×10^{-57}
rs10122541	9q22.33	G	1.54	1.40	1.70	1.10×10^{-17}
rs7037324	9q22.33	A	1.54	1.39	1.70	1.20×10^{-17}
rs7902587	10q24.33	T	1.41	1.27	1.56	5.40×10^{-11}
rs368187	14q13.3	G	1.39	1.30	1.47	5.10×10^{-23}
rs944289	14q13.3	T	1.35	1.27	1.44	2.10×10^{-19}
rs116909374	14q13.3	T	1.71	1.47	2.00	6.20×10^{-12}
rs10136427	14q24.3	C	1.40	1.23	1.60	4.35×10^{-07}
rs2289261	15q22.33	C	1.23	1.15	1.32	3.10×10^{-09}
rs56062135	15q22.33	T	1.24	1.16	1.34	4.90×10^{-09}
rs7267944	20q12	C	1.39	1.24	1.56	2.13×10^{-08}

SNP, single nucleotide polymorphism; OR, odds ratio; LCI, lower confidence interval; UCI, upper confidence interval

Table S3. Characteristics of GWAS summary data for socioeconomic factors and thyroid cancer in European ancestry

**Phenotype**	**GWAS ID**	**Year**	**Sample size**	**No. SNPs**	**Author**	**Consortium**
Thyroid cancer	finn-b-C3_THYROID_GLAND_EXALLC	2023	288,920	18,707,521	NA	FinnGen
Average total household income	ukb-b-7408	2018	397,751	9,851,867	Elsworth B	MRC-IEU
College or University degree	ukb-a-397	2017	334,070	9,851,867	Neale	Neale Lab
Unemployed status	ukb-b-12885	2018	461,242	9,851,867	Elsworth B	MRC-IEU
Frequency of friend/family visits	ukb-b-5379	2018	459,830	9,851,867	Elsworth B	MRC-IEU

GWAS, genome-wide association study; SNP, single nucleotide polymorphism
